# Mortality of trauma patients treated at trauma centers compared to non-trauma centers in Sweden: a retrospective study

**DOI:** 10.1007/s00068-020-01446-6

**Published:** 2020-07-27

**Authors:** Stefan Candefjord, Linn Asker, Eva-Corina Caragounis

**Affiliations:** 1 Department of Electrical Engineering, Chalmers University of Technology and MedTech West, Sahlgrenska University Hospital, Gothenburg, Sweden; 2 Department of Surgery, Institute of Clinical Sciences, Sahlgrenska University Hospital, Sahlgrenska Academy, University of Gothenburg, Per Dubbsgatan 15, SE 413 45 Gothenburg, Sweden

**Keywords:** Trauma, Triage, Trauma center, Trauma system, Mortality, SweTrau

## Abstract

**Objective:**

The main objective was to compare the 30-day mortality rate of trauma patients treated at trauma centers as compared to non-trauma centers in Sweden. The secondary objective was to evaluate how injury severity influences the potential survival benefit of specialized care.

**Methods:**

This retrospective study included 29,864 patients from the national Swedish Trauma Registry (SweTrau) during the period 2013–2017. Three sampling exclusion criteria were applied: (1) Injury Severity Score (ISS) of zero; (2) missing data in any variable of interest; (3) data falling outside realistic values and duplicate registrations. University hospitals were classified as trauma centers; other hospitals as non-trauma centers. Logistic regression was used to analyze the effect of trauma center care on mortality rate, while adjusting for other factors potentially affecting the risk of death.

**Results:**

Treatment at a trauma center in Sweden was associated with a 41% lower adjusted 30-day mortality (odds ratio 0.59 [0.50–0.70], *p* < 0.0001) compared to non-trauma center care, considering all injured patients (ISS ≥ 1). The potential survival benefit increased substantially with higher injury severity, with up to > 70% mortality decrease for the most critically injured group (ISS ≥ 50).

**Conclusions:**

There exists a potentially substantial survival benefit for trauma patients treated at trauma centers in Sweden, especially for the most severely injured. This study motivates a critical review and possible reorganization of the national trauma system, and further research to identify the characteristics of patients in most need of specialized care.

## Introduction

Trauma is a major cause of mortality, responsible for 9% of global deaths and the primary reason for loss of life in young people [1]. Many deaths are preventable with more effective and fast treatment [2, 3]. This can be accomplished by streamlining the prehospital chain and, with minimum delay, provide treatment at specialized trauma centers [4–6]. Trauma systems vary considerably in different parts of the world, with diverse policies concerning transport destination of the injured patient. International studies indicate that treating severely injured patients at trauma centers, which are better equipped to provide adequate care, is associated with a reduced mortality of 15–32% [4–8]. In many countries, the policy is to transport severely injured patients directly to trauma centers, even if it means bypassing lower level care facilities in closer proximity [4, 7, 9, 10].

Prehospital undertriage occurs when a severely injured patient, Injury Severity Score [11] (ISS) > 15, is transported to a facility that lacks the required level of appropriate care, and should be less than 5% [10]. Unfortunately, the rate of undertriage is much higher. Estimations from studies in the US and Europe indicate rates around 20–60% [12–17]. In contrast, overtriage, whereby minimally injured patients are transported to higher level trauma centers, should be restricted to 25–35% [10]. Possible reasons for triage errors include limited sensitivity of triage protocols in predicting severe injury [18, 19], suboptimal adherence to triage protocols and transportation policies [20, 21], and difficulties with recognizing occult injuries in the field [22, 23].

The fact that a large proportion of major trauma patients do not receive adequate care leads to high rates of preventable deaths. Some studies suggest that the potentially preventable death rate is around 20–40% [2, 3, 24]. In Sweden, a high proportion of major trauma patients and the majority of patients sustaining severe injury in motor vehicle crashes are transported to non-trauma centers, indicating a high rate of undertriage [12, 13]. The predominant reason may be that most regional guidelines do not support bypassing the closest care facility in favor of a more distant regional trauma center [12]. In Scandinavia, due to sparse population density and long duration of transportation, trauma care relies more on organized inter-hospital transfer [25]. In fact, distance to the nearest trauma center has the strongest influence on the transport destination decision, whereby the odds of being transported to a trauma center decreases with 5% for every kilometer [13]. No national study has previously evaluated if the high rate of undertriage leads to a difference in mortality rate between Sweden’s regional trauma centers, the University Hospitals, and non-trauma centers.

This study aims to investigate the potential survival benefit for trauma patients treated at regional trauma centers in Sweden. The main objective is to compare the 30-day mortality rate of trauma patients treated at trauma centers as compared to non-trauma centers. The secondary objective is to evaluate how injury severity influences the potential survival benefit of specialized trauma care.

## Methods

### Study design and setting

This was a retrospective cohort study that evaluated potential differences in mortality between trauma patients treated at regional trauma centers and other trauma care facilities in Sweden. Data on trauma patients are reported into the Swedish Trauma Registry (SweTrau). SweTrau is the only nationwide trauma database, covering 84% [26] of the trauma receiving hospitals in Sweden in 2017. The SweTrau database follows the *“The Utstein Trauma Template for Uniform Reporting of Data following Major Trauma: Data Dictionary”,* which represents a uniform set of variables considered most important for comparing trauma systems and outcomes in Europe [27, 28]. To be registered in SweTrau, patients have to fulfil at least one inclusion criteria; Trigger a trauma team activation, and/or have a New Injury Severity Score [29] (NISS) > 15, and/or have a NISS > 15 and be transferred from another hospital within 7 days. Trauma team activations are either Level 1 triggers whereby a large team resuscitates trauma patients with physiological impairment with or without obvious injury, or Level 2 triggers whereby a limited team assesses stable trauma patients subjected to specific mechanisms of injury [30]. Patients who trigger trauma team activation without a traumatic event or have an isolated chronic subdural hematoma are excluded from the registry. Only patients arriving to the hospital alive are registered.

A statistical power analysis was performed before the study commenced. The study should be powered to detect a 3% difference or larger in mortality between trauma centers and non-trauma centers. To demonstrate a 3% difference with statistical significance of 5% and statistical power of 80%, inclusion of approximately 2500 severely injured (ISS > 15) patients was needed [31].

### Trauma care in Sweden

**Fig. 1:**
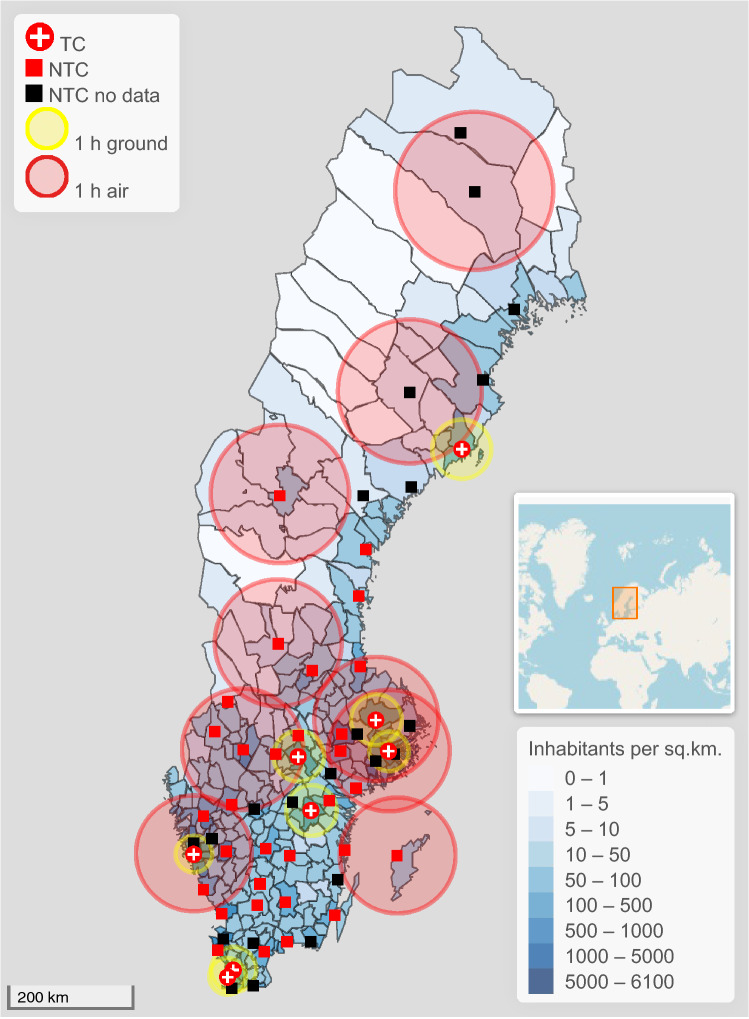
Map of Sweden showing the population density for each of Sweden’s 290 municipalities (inhabitants/km^2^, intervals closed at start, open at end) and estimated 1-h radii for transport to a trauma center or non-trauma center (round trip for prehospital team), with an ambulance helicopter 107 km (red circles) or ground ambulance 45 km for smaller cities and 35 km for larger cities (yellow circles). Trauma-receiving hospitals not reporting to SweTrau within the queried time period are shown in black. The southernmost trauma center, Skåne University Hospital, consists of two hospitals in two cities in close proximity, shown individually. For increased clarity, a few hospitals close to trauma centers have been omitted, and no 1-h radii for ground transportation is shown for non-trauma centers. Estimated 1-h radii only take round trip transportation time into account, no allowance has been made for other factors such as time spent on site, loading and offloading patients, traffic or weather conditions or the starting point for the prehospital team. The map provides an approximative visualization of the trauma care conditions in Sweden. The minimap shows the location of Sweden

Sweden is overall a sparsely inhabited country with a population of just over 10.4 million people covering an area of approximately 420,000 km^2^. This produces a population density of 25 people/km^2^ on average; however, 87% of the population live on 1.5% of the entire land area (SCB, Statistics Sweden, www.statistikdatabasen.scb.se, accessed 2020-05-16). In general, people that live in metropolitan areas have quick access, within 1 h either by ground transport or helicopter, to a University Hospital (Fig. 1). However, outside metropolitan areas quick access to trauma centers is generally not provided. There are nine ambulance helicopters containing a team of anesthetic doctor and nurse (Fig. 1). However, only three University Hospitals have access to their own helicopter. The remaining six helicopters are mainly active in more sparsely inhabited areas. Figure 1 shows the 1-h radius for transport time for all ambulance helicopters, and 1-h radii for ground ambulances to trauma centers. The transport time was estimated for a prehospital team leaving the hospital and collecting the patient to the same hospital (107 km for helicopters, 45 km for ambulances in small cities/towns, and 35 km for ambulances in large cities). The estimates do not account for time spent on site, loading and offloading patients, traffic or weather conditions, or that the prehospital team may be in close proximity to the patient at the time of alert. The 1-h radii can, therefore, be both longer and shorter, and the map should be seen as a rough estimation. There is no national trauma system in Sweden. The country is divided into 21 regions, and each region has a designated University Hospital, seven in total. The University Hospitals serve as regional trauma centers, as they are the only hospitals with neurosurgical capabilities among other sub-specialties. Each region develops its trauma care individually. Non-trauma centers may differ in the level of care that they can provide. However, the hospitals reporting into SweTrau are generally the larger hospitals with more equal capabilities. The trauma patient load varies between the regional trauma centers and may be less than that required of a Level I trauma center when comparing to conditions in the US [10]. According to data from SweTrau (2013–2017), 69% of patients with ISS > 15 are treated at trauma centers, on average 734 patients/year, and 31% at non-trauma centers, on average 331 patients/year.

### Selection of participants

All hospitals reporting to SweTrau during the period 2013–2017 were included, in total 42 hospitals whereof seven University Hospitals, serving as regional trauma centers. Figure 1 shows the locations of the included hospitals, and locations for trauma-receiving hospitals not reporting data to SweTrau. The queried time period was chosen to obtain a large dataset with broad national coverage of hospitals. The completion of registering patients in SweTrau is lagging and 2017 was the most recent year with complete data available when this study commenced. Data on all patients, both children and adults, registered in SweTrau during the study period were extracted. We applied three sampling exclusion criteria: (1) no registered injury, i.e. ISS = 0; (2) missing data in any variable of interest for the primary analysis; (3) data falling outside realistic values and duplicate registrations.

### Measurements and outcomes

The variables of interest were selected from known potential risk factors for mortality identified from the literature [4, 32–37]. They were included as model covariate variables to adjust the estimated mortality rates for the logistic regression models. Care was taken to not include two/several variables with strong correlation, e.g. ISS and NISS, in logistic regression modelling. The selected variables of interest were sex, age, hospital (trauma center or non-trauma center), dominant type of injury (blunt or penetrating), ISS, transfer (direct or transferred), transportation time, and Glasgow Coma Scale assessed at the emergency department. The 30-day mortality outcome was used as the dependent variable in the logistic regression models. Deaths occurring later than 30 days following injury, and foreign citizens alive when repatriated to their home country within 30 days, were coded as survivors [27].

### Data analysis

The data were carefully reviewed to check variable distributions and identify extreme values, incorrect values, duplicate registrations, and unknown or missing data. Descriptive statistics of patient and injury characteristics along with treatment data and outcome measures were reported as number and percentages for four patient groups: (1) patients excluded from the primary analysis after applying the sampling exclusion criteria; (2) patients included in the primary analysis; (3) included patients treated at a trauma center; (4) included patients treated at a non-trauma center. Baseline characteristics between group 1 and group 2, and between group 3 and group 4, were derived for the most relevant data for this study. Unknown and missing data were combined and denoted as “Unknown”. Pearson’s Chi square test was used to check for statistically significant differences between the observed frequencies in all categories of each variable, followed by a Z-test to detect significant differences between the observed frequency of each variable category. Mood’s median test was used to test for significant differences in median values.

Selected variables of interest that were continuous or ordinal (age, ISS, transportation time, and Glasgow Coma Scale) were coded as categorical variables. Age was divided into the groups 0–15 (pediatric), 16–45 (young adults), 46–60 (middle aged), 61–75 (old adults), and > 75 years (geriatric), as different age groups have different susceptibility and vulnerability to trauma [37, 38]. Transportation time was divided into 0–20 (short), 20–45 (medium), 45–90 (long), and > 90 min (prolonged), as patients were categorized as injured in the hospital vicinity (short) and with increasingly longer transportation time (medium, long, prolonged). For ISS categorization, a modification of Cope’s categories [39] was employed to form the categories 1–8, 9–15, 16–24, 25–49 and 50–75. Glasgow Coma Scale scores were divided into three groups of increasing head injury severity: 13–15 (minor); 9–12 (moderate); 3–8 including patients who were intubated (severe) [40, 41]. Patients were categorized as undergoing direct transfer to reporting hospital or categorized as transferred if they received treatment in more than one hospital.

The study’s primary objective was assessed using the selected variables of interest as predictor variables in a binomial logistic regression model with 30-day mortality as dependent variable. The procedure tested the null hypothesis that there was no difference in mortality between patients cared for at trauma centers compared to non-trauma centers, after adjusting for other potential risk factors for death. The primary analysis included all patients after applying the three sampling exclusion criteria. The study’s secondary objective was assessed by performing logistic regression analyses analogous to the primary analysis, but restricting inclusion according to different ISS levels. Five logistic regression models were constructed for the secondary analysis, by forming the groups 1 ≤ ISS ≤ 8, ISS ≥ 9, ISS ≥ 16, ISS ≥ 25, and ISS ≥ 50. The same variables of interest as for the primary analysis were used and modeled in the same way, except for that ISS was modeled as a continuous variable when only a single ISS category was included. Results from the primary logistic regression analysis were presented by *p*-values to evaluate the statistical significance of each variable category, and odds ratios with 95% confidence intervals for each variable category as compared to the chosen reference level. Results from the secondary analysis was presented as *p*-value and odds ratio with 95% confidence interval for the hospital variable (trauma center vs non-trauma center). The logistic regression models were assessed for adequate fit to data by verifying statistical significance using the log-likelihood ratio test, and by evaluating McFadden’s pseudo R^2^ value for the model. All data analyses were performed using Python (version 3.7.4) and the libraries pandas (0.25.1), SciPy (1.3.1) and Statsmodels [42] (version 0.10.1). Histograms were plotted using R (version 3.6.1). The population density plot was plotted using R and the Leaflet library (version 2.0.2). A *p*-value less than 0.05 was considered statistically significant.

## Results

### Characteristics of study subjects

**Fig. 2:**
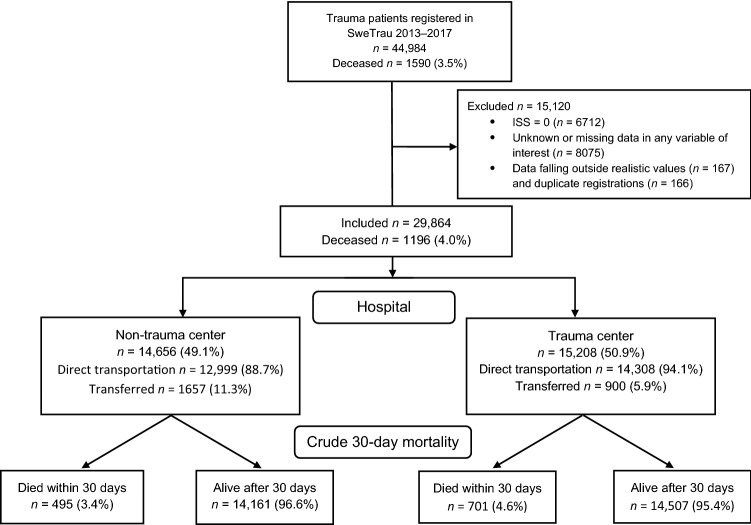
Flow-chart for patient selection

There were 44,984 patients registered in SweTrau 2013–2017. Applying the three sampling criteria resulted in a sample of 29,864 patients for the primary analysis. Excluded patients were younger, less injured, and arrived at the hospital more often by alternative means (Table 1). There was a higher percentage of females, non-trauma center patients and transferred patients in this group. The flowchart for final patient selection is presented in Fig. 2. Out of the included patients, 3968 were severely injured (ISS > 15) (Table 1), which surpassed the number required from the power analysis of 2500 patients.

**Table 1:** Descriptive statistics of the study population (*n* = 44,984) presented in four different groups from left to right: (1) Patients excluded from the main analysis; (2) Patients included in the main analysis; (3) Included patients treated at a non-trauma center (NTC); (4) Included patients treated at a trauma center (TC)

Variable	Levels	Excluded	Included	NTC	TC
Total nr of registrations	–	15,120	29,864	14,656	15,208
Sex^ab^	Male	9503 (62.9)*	19,356 (64.8)	9276 (63.3)*	10,080 (66.3)
	Female	5569 (36.8)*	10,508 (35.2)	5380 (36.7)*	5128 (33.7)
	Unknown	48 (0.3)*	0 (0.0)	0 (0.0)	0 (0.0)
Age (years)^ab^	0–15	2010 (13.3)*	3442 (11.5)	1726 (11.8)	1716 (11.3)
	16–45	7768 (51.4)*	14,231 (47.7)	6816 (46.5)*	7415 (48.8)
	46–60	2501 (16.5)*	5559 (18.6)	2642 (18.0)*	2917 (19.2)
	61–75	1788 (11.8)*	3902 (13.1)	2042 (13.9)*	1860 (12.2)
	76–107	960 (6.3)*	2730 (9.1)	1430 (9.8)*	1300 (8.5)
	Unknown	93 (0.6)*	0 (0.0)	0 (0.0)	0 (0.0)
Dominant type of injury^ab^	Blunt	13,153 (87.0)*	27,517 (92.1)	13,863 (94.6)*	13,654 (89.8)
	Penetrating	722 (4.8)*	2347 (7.9)	793 (5.4)*	1554 (10.2)
	Unknown	1245 (8.2)*	0 (0.0)	0 (0.0)	0 (0.0)
Mechanism of injury^ab^	Motor vehicle crash	5372 (35.5)*	8548 (28.6)	5117 (34.9)*	3431 (22.6)
	Motorcycle	1052 (7.0)*	2325 (7.8)	1059 (7.2)*	1266 (8.3)
	Bicycle	951 (6.3)*	2411 (8.1)	1137 (7.8)*	1274 (8.4)
	Pedestrian	351 (2.3)*	1027 (3.4)	346 (2.4)*	681 (4.5)
	Traffic other	299 (2.0)*	493 (1.7)	339 (2.3)*	154 (1.0)
	Shot	182 (1.2)	415 (1.4)	74 (0.5)*	341 (2.2)
	Stab	462 (3.1)*	1657 (5.5)	492 (3.4)*	1165 (7.7)
	Blunt object	711 (4.7)*	1895 (6.3)	679 (4.6)*	1216 (8.0)
	Low fall < 3 m	1215 (8.0)*	3312 (11.1)	1426 (9.7)*	1886 (12.4)
	High fall > 3 m	2709 (17.9)*	6501 (21.8)	3111 (21.2)*	3390 (22.3)
	Explosion	43 (0.3)	70 (0.2)	40 (0.3)	30 (0.2)
	Other	595 (3.9)	1134 (3.8)	805 (5.5)*	329 (2.2)
	Unknown	1178 (7.8)*	76 (0.3)	31 (0.2)	45 (0.3)
ISS^ab^	0	6712 (44.4)	0 (0.0)	0 (0.0)	0 (0.0)
	1–8	5101 (33.7)*	20,470 (68.5)	10,669 (72.8)*	9801 (64.4)
	9–15	1496 (9.9)	5426 (18.2)	2477 (16.9)*	2949 (19.4)
	16–24	889 (5.9)*	2156 (7.2)	886 (6.0)*	1270 (8.4)
	25–49	848 (5.6)*	1570 (5.3)	570 (3.9)*	1000 (6.6)
	50–75	73 (0.5)	242 (0.8)	54 (0.4)*	188 (1.2)
	Unknown	1 (0.0)	0 (0.0)	0 (0.0)	0 (0.0)
Type of transport	Ground ambulance	8584 (56.8)*	27,645 (92.6)	14,006 (95.6)*	13,639 (89.7)
	Helicopter ambulance	230 (1.5)*	2195 (7.3)	637 (4.3)*	1558 (10.2)
	Fixed-wing ambulance	1 (0.0)	10 (0.0)	7 (0.0)	3 (0.0)
	Private/public vehicle	2202 (14.6)*	1 (0.0)	1 (0.0)	0 (0.0)
	Walk-in	1421 (9.4)*	0 (0.0)	0 (0.0)	0 (0.0)
	Police	153 (1.0)*	0 (0.0)	0 (0.0)	0 (0.0)
	Other	114 (0.8)*	0 (0.0)	0 (0.0)	0 (0.0)
	Not applicable	1772 (11.7)*	6 (0.0)	2 (0.0)	4 (0.0)
	Unknown	643 (4.3)*	7 (0.0)	3 (0.0)	4 (0.0)
Transportation time (min)^ab^	0–20	5223 (34.5)*	18,176 (60.9)	7145 (48.8)*	11,031 (72.5)
	20–45	2680 (17.7)*	9253 (31.0)	5377 (36.7)*	3876 (25.5)
	45–90	642 (4.2)*	2260 (7.6)	1979 (13.5)*	281 (1.8)
	> 90	97 (0.6)	175 (0.6)	155 (1.1)*	20 (0.1)
	Unknown	6478 (42.8)*	0 (0.0)	0 (0.0)	0 (0.0)
Hospital types^a^	NTC	8901 (58.9)*	14,656 (49.1)	14,656 (100.0)	0 (0.0)
	TC	6219 (41.1)*	15,208 (50.9)	0 (0.0)	15,208 (0.0)
	Unknown	0 (0.0)	0 (0.0)	0 (0.0)	0 (0.0)
Transfer^ab^	Direct	11,528 (76.2)*	27,307 (91.4)	12,999 (88.7)*	14,308 (94.1)
	Transferred	2372 (15.7)*	2557 (8.6)	1657 (11.3)*	900 (5.9)
	Unknown	1220 (8.1)*	0 (0.0)	0 (0.0)	0 (0.0)
GCS^ab^	13–15 (minor)	10,142 (67.1)*	27,347 (91.6)	13,817 (94.3)*	13,530 (89.0)
	9–12 (moderate)	194 (1.3)*	876 (2.9)	288 (2.0)*	588 (3.9)
	3–8 + intubated (severe)	943 (6.2)*	1641 (5.5)	551 (3.8)*	1090 (7.2)
	Unknown	3841 (25.4)*	0 (0.0)	0 (0.0)	0 (0.0)
Highest level of care^ab^	Emergency department	7282 (48.2)*	11,410 (38.2)	5998 (40.9)*	5412 (35.6)
	General ward	2584 (17.1)*	7697 (25.8)	3976 (27.1)*	3721 (24.5)
	Operation theatre	626 (4.1)*	2207 (7.4)	842 (5.7)*	1365 (9.0)
	High dependency unit	951 (6.3)*	3315 (11.1)	1159 (7.9)*	2156 (14.2)
	ICU	2480 (16.4)*	5234 (17.5)	2680 (18.3)*	2554 (16.8)
	Unknown	1197 (7.9)*	1 (0.0)	1 (0.0)	0 (0.0)
Mortality 30-day^ab^	Dead	394 (2.6)*	1196 (4.0)	495 (3.4)*	701 (4.6)
	Alive	13,126 (86.8)*	28,668 (96.0)	14,161 (96.6)*	14,507 (95.4)
	Unknown	1600 (10.6)*	0 (0.0)	0 (0.0)	0 (0.0)

The data are presented as number of cases and in column percentages. Statistical comparisons have been made between the excluded and included group, and the NTC and TC groups, respectively

*ISS* Injury Severity Score, *GCS* Glasgow Coma Scale, *ICU* Intensity care unit

^a^ Statistical significance with Person Chi Square test, comparing column proportions between excluded and included patients

*Statistical significance with Z-test, comparing pairwise column proportions between excluded and included patients (denoted in column “Excluded”) and NTC and TC (denoted in column “NTC”), respectively

^b^ Statistical significance with Person Chi Square test, comparing column proportions between NTC and TC

### Main results

Descriptive statistics for the included patients are presented in Table 1. Crude 30-day mortality was 4.6% for patients treated at trauma centers compared to 3.4% for patients treated at non-trauma centers. Note that some patients were included more than once due to having experienced two or several traumatic events during the queried time period, so the total number of unique patients was less than the total number of registrations. Median age was 38 (10th and 90th percentiles, [*P*_*10*_*, P*_*90*_] = [15, 73]) and 38 ([*P*_*10*_*, P*_*90*_] = [15, 75]) (*p* > 0.05) at trauma centers and non-trauma centers, respectively. There was a higher proportion of patients < 60 years of age at trauma centers. Patients managed at trauma centers were more severely injured with a higher ISS and lower GCS score, and with a higher prevalence of males and penetrating trauma. Median ISS was 5 (10th and 90th percentiles, [*P*_*10*_*, P*_*90*_] = [1, 21]) and 4 ([*P*_*10*_*, P*_*90*_] = [1, 16]) (*p* < 0.0001) at trauma centers and non-trauma centers, respectively.

**Fig. 3:**
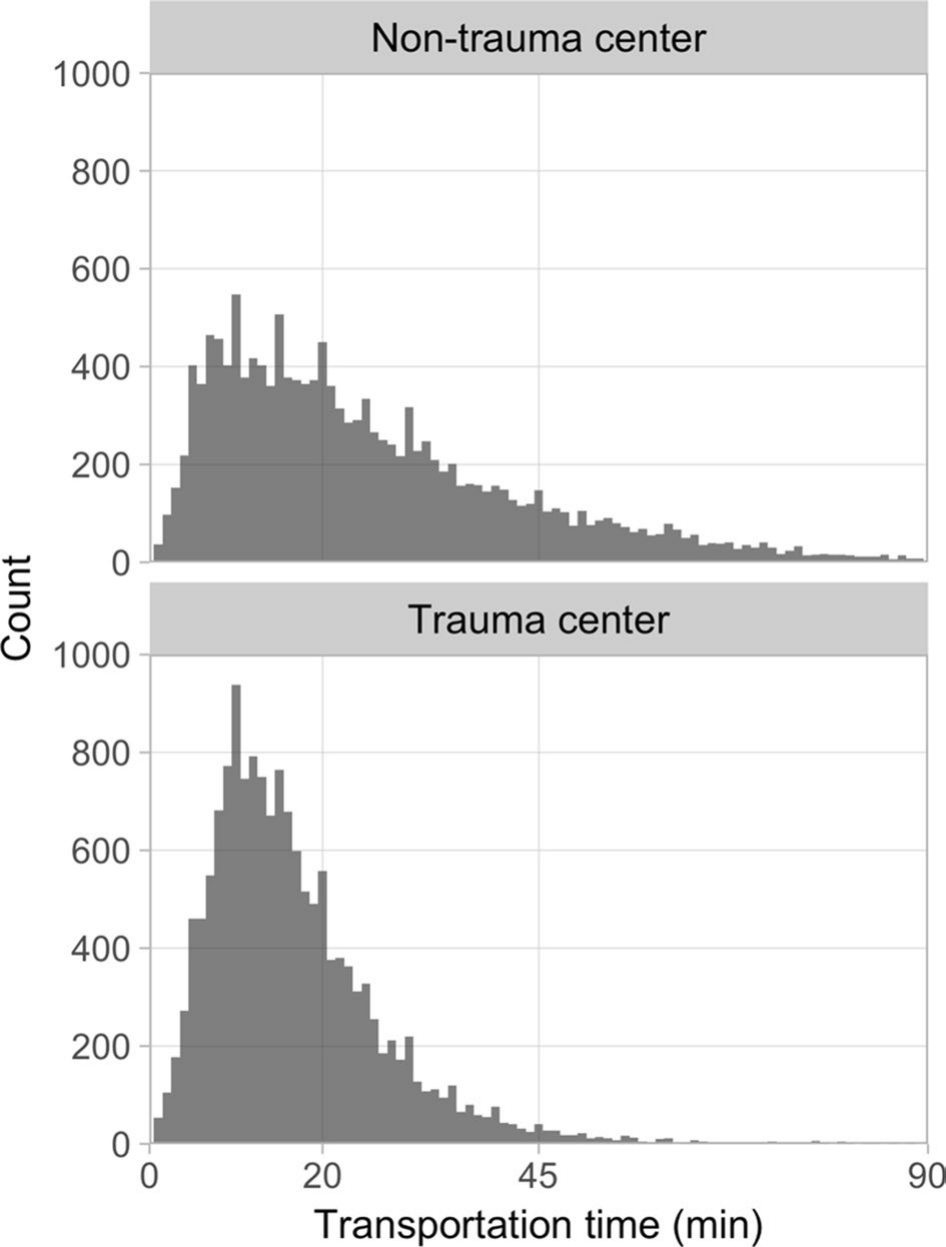
Histograms of the transportation time for non-trauma center patients (top) and trauma center patients (bottom). The histograms were limited at *x* = 90 min for increased clarity and the fact that few patients (*n* = 175) had transportation times > 90 min

Patients were mainly transported by ground ambulance. Helicopter transport was more common in patients admitted to trauma centers (Table 1). The transportation time was shorter for patients admitted to trauma centers, and there was a larger variability of transportation time for patients received at non-trauma centers (Fig. 3). Median transport time was 15 min ([*P*_*10*_*, P*_*90*_] = [6, 30]) and 21 min ([*P*_*10*_*, P*_*90*_] = [7, 53]) (*p* < 0.0001) for trauma center patients and non-trauma center patients, respectively. The majority of patients were directly transported to the hospital where they acquired definitive care; however, there was a larger proportion of transferred patients at non-trauma centers. Level 1 trauma team activations were triggered in 54% of registrations, at both trauma centers and non-trauma centers. Level 2 trauma team activations were triggered in 36% vs 32% of registrations (*p* < 0.0001) at trauma centers and non-trauma centers, respectively. There was a higher proportion of no trauma team activation at non-trauma centers. Patients admitted to trauma centers required operative intervention to a larger degree compared to patients admitted to non-trauma centers (Table 1).

Table 2 shows the results from the primary analysis, i.e. the logistic regression model for the patients with ISS ≥ 1 (*n* = 29,864). The odds ratio for treatment at a trauma center was 0.59 [0.50, 0.70], and the null hypothesis that there was no difference in mortality between trauma centers and non-trauma centers was rejected (*p* < 0.0001). In addition, increasing age and ISS, decreasing GCS and penetrating trauma were predictive of mortality. Longer transportation time did not increase mortality. There was no statistically significant difference between short, medium and prolonged transportation times. The transferal of patients and a transportation time within the period 45–90 min seemed to have a protective effect.

**Table 2:** Result of logistic regression including all injured patients (ISS ≥ 1)

Variable/levels	*p*-value	Odds ratio [95% CI]
Sex/male		
Female	0.081	0.86 [0.72, 1.0]
Age group (0–15)		
16–45	0.69	0.90 [0.55, 1.5]
46–60	**0.025**	1.8 [1.1, 3.0]
61–75	**<** **0.0001**	4.7 [2.9, 7.7]
> 76	**<** **0.0001**	27 [17, 44]
Hospital (non-trauma center)		
Trauma center	**<** **0.0001**	0.59 [0.50, 0.70]
Dominant type of injury (blunt)		
Penetrating	**<** **0.0001**	2.6 [2.0, 3.5]
ISS (1–8)		
9–15	**<** **0.0001**	2.6 [2.0, 3.2]
16–24	**<** **0.0001**	4.9 [3.8, 6.4]
25–49	**<** **0.0001**	21 [17, 27]
50–75	**<** **0.0001**	72 [48, 110]
Transfer (direct)		
Transferred	**<** **0.0001**	0.26 [0.20, 0.33]
Transportation time (0–20)		
20–45	0.84	0.98 [0.82, 1.2]
45–90	**0.0017**	0.52 [0.35, 0.78]
> 90	0.69	1.2 [0.45, 3.3]
Glasgow Coma Scale (Minor)		
Moderate	**<** **0.0001**	4.5 [3.4, 6.0]
Severe	**<** **0.0001**	25 [20, 31]
Model intercept	**<** **0.0001**	0.0024 [0.0015, 0.0040]

*CI* confidence interval

*p-*values* < *0.05 are highlighted in bold

The results from the secondary analysis are presented in Table 3. There was no statistically significant difference in mortality between trauma patients in trauma centers and non-trauma centers in the group with no serious injury (AIS < 3), 1 ≤ ISS ≤ 8. For patients with ISS ≥ 9, treatment at trauma centers was associated with a statistically significant decrease in mortality (*p* < 0.0001). With increasing injury severity, the seemingly protective effect of specialized trauma care increased (*p* < 0.0001), up to > 90% for the most critically injured. Note that for the group ISS ≥ 50, the number of patients was relatively small and the estimated odds ratio was more uncertain with a relatively large confidence interval.

**Table 3:** Results for the hospital variable (trauma center versus non-trauma center) for separate logistic regression models of different patient groups with increasing injury severity

Patient group	Nr of pat	TC *p*-value	TC odds ratio [95% CI]
1 ≤ ISS ≤ 8^a^	20,470	0.26	0.81 [0.56, 1.2]
ISS ≥ 9	9394	**<** **0.0001**	0.54 [0.44, 0.65]
ISS ≥ 16	3968	**<** **0.0001**	0.44 [0.35, 0.56]
ISS ≥ 25	1812	**<** **0.0001**	0.28 [0.21, 0.39]
ISS ≥ 50^a^	242	**0.0012**	0.043 [0.0064, 0.29]

The predictor variables were unchanged, except that ISS was modelled as a continuous variable when only a single ISS group category was included in the analysis. Non*-*trauma center was used as the reference variable for all analyses. *p-*values* < *0.05 are highlighted in bold

*Nr of pat* number of patients, *TC* trauma center, *CI* confidence interval

^a^ISS was analysed as a continuous variable in the logistic regression

We observed quality issues with the *Transfer* variable, which only states that the patient has been transferred to or from another hospital but not whether the transfer was from a lower level care, higher level or the same level facility. This brought that we could not clearly follow the complete inter-hospital transfer chain for individual traumatic events. Therefore, the analyses were repeated with the exclusion of transferred patients, with very similar results (data not shown), demonstrating that the analysis was not biased due to inclusion of transferred patients.

Since a large number of cases were removed due to having no apparent injury (ISS = 0) or missing data (Fig. 2), we performed additional analyses where missing data were imputed using the strategies to replace missing values with the median value for continuous variables and the mode (most frequent value) for categorical variables. 37,538 cases were thus retained (still excluding patients with ISS = 0, patients with unknown status of mortality, observations with unrealistic values and duplicate registrations). The trends were similar to the analyses using only complete cases. Treatment at a trauma center was associated with a decreased mortality with an odds ratio of 0.53 [0.46, 0.62] (*p* < 0.0001). In general, the association of trauma center care and decreased risk of mortality was stronger when estimated using the imputed data set. The only exception was for patients with ISS ≥ 50, for which the odds ratio was 0.28 [0.10, 0.76] for the imputed data set.

## Discussion

This is the first national study in Sweden to compare mortality between patients managed at University Hospitals, functioning as regional trauma centers, with non-trauma centers. By including data from the national trauma registry, SweTrau, during a 5-year period, a sample of almost 30,000 patients with complete data on relevant variables was compiled. Estimations on the effect of specialized care was derived after adjusting for possible confounding factors. Patients treated at trauma centers were not comparable to patients treated at non-trauma centers, as these were younger and more seriously injured. However, after adjustment, we found that trauma center care was associated with decreased mortality compared to non-trauma center care, especially for the most critically injured patients, and this difference was larger than what has been reported in comparable international studies [4, 6, 33]. Trauma center care was associated with a 41% lower adjusted 30-day mortality rate. The results for the main logistic regression model was in general in good agreement with findings reported in the literature [5, 6, 43].

Patients lacking a serious injury did not seem to have a survival benefit of being managed at a trauma center. Decreased mortality was seen in patients with ISS ≥ 9 managed at a trauma center. Although the SweTrau registry uses NISS [29], as this has been shown to better indicate severity of injury and be a better predictor of mortality [44], we chose to study ISS [11] in our logistic regression models. The reason for this being the vast body of research utilizing ISS to compare our results to those from other studies. It has been suggested that ISS > 12 should be used as a cut-off for a severely injured patient instead of ISS > 15 [45]. However, we chose to use the traditional Cope’s categories [39].

The age-dependent increase in mortality is in agreement with published studies, e.g. Kojima et al. [43] found odds ratios of 1.60–6.09 for age intervals 60–69, 70–79, 80–89 and ≥ 90, although a more pronounced effect was observed for the oldest patients in the present study (odds ratio 31 with 95% confidence interval [19, 53]).

From the study by Fagerlind et al. [13], it is known that the distance to trauma center has a strong influence on the transport decision in the Swedish prehospital care setting. A difference in transportation times between patients managed at trauma centers compared to non-trauma centers was, therefore, anticipated, and confirmed by statistical analysis (Table 1). The variability of transportation time was visualized in histograms (Fig. 3). A systematic difference in transportation times may affect mortality and constitute a confounding variable. It was, therefore, deemed important to include transportation time as a variable in the main analysis using logistic regression modelling. We found that a transportation time of 20–45 min did not contribute to increased mortality as compared to the reference level 0–20 min (Table 2). The fact that increased transportation time was not associated with increased mortality is in agreement with other studies that have found no association between mortality and prehospital time ≥ 60 min [46, 47], except for in patients in shock requiring critical intervention [47, 48]. However, in our study, patients with a prehospital transportation time interval between 45 and 90 min had lower mortality as compared to the reference level. The reason for this is unknown. A speculation is that patients within this group were considered by the prehospital staff as not suffering from potentially lethal injuries and being able to tolerate longer transportation times, manifesting as a protective effect in our analysis. A similar survival bias has been seen in other studies [49, 50]. Furthermore, the type of transport was not studied in relation to transportation time. It is possible that patients transported for a longer time were in a helicopter with a higher level of care prehospitally, since these are manned by anesthetic doctors and nurses, and with better resuscitation as they may have blood available for transfusion. However, the results indicate that transportation time may not be as important as choosing the right destination for the patient. We should also remember that only patients arriving alive at the hospital were included in the registry, so it is possible that patients with severe injuries and long transportation times died *en route*. However, there were too few patients in the category ISS ≥ 50 to draw conclusions concerning what can be considered to be an acceptable transportation time in this group.

Analyzing specific injuries was outside the scope of this study. It is conceivable that differences in the type and severity of injury could influence the differences in mortality between trauma centers and non-trauma centers. Especially, differences in neurosurgical emergencies could bias the results. Although we did not study injury patterns, we included GCS and ISS in our regression model, which would covariate with maximum AIS and with severe traumatic brain injury. Future studies can be aimed at identifying which specific injures that are best treated at trauma centers and non-trauma centers, respectively [32]. We found that patients without serious injuries (AIS < 3) and ISS < 9 have equal mortality rate regardless of whether they are transported to a trauma center or a non-trauma center. In contrast, trauma center care is associated with a decreasing mortality with increasing ISS for patients with ISS ≥ 9.

We found that trauma center patients underwent operative intervention to a greater extent, as compared to non-trauma center patients. Severely injured trauma patients may in some instances require care at a trauma center to survive. For these patients, a comparison concerning survival between trauma center and non-trauma center care is unrealistic, as they all require trauma center care in order to survive. To study and compare these cases, it would also have been beneficial to have access to prehospital vital signs. Unfortunately, prehospital triage criteria could not be evaluated as prehospital variables contained large proportions of missing/unknown data concerning vital signs.

This study has several limitations. The results are subject to the retrospective nature of the data found in the Swedish national trauma registry, SweTrau. This is the only national injury database covering all mechanisms of injury; however, registering in SweTrau is voluntary. Although an increasing number of hospitals report to SweTrau, 84% in 2017 [26], the patient coverage rate is unknown. By comparing trauma patients in the national Intensive Care Unit (ICU) register, Svenska Intensivvårdsregistret (SIR), to the number of patients requiring ICU care in SweTrau, an estimated 81% of all trauma patients are included [26]. Regardless of possible overestimation concerning coverage and the problem with delayed reporting, it is unlikely that a systematic bias exists whereby mortalities are included to a greater extent at neither a trauma center nor a non-trauma center. Quality issues were observed with the registration procedure. They consisted of some duplicate registrations of the same traumatic event at the same hospital, a few entries with unrealistic values of certain variables, and a relatively large number of unknown or missing data. As a consequence, many patients needed to be excluded from our main analysis (Fig. 2), which limited the precision in estimating effect sizes and possibly biased results, although statistical power was reached. The additional analyses using imputation by median and mode for missing data confirmed the potential protective effect of trauma center care. The odds ratios for care at trauma center were lower compared to the complete case analyses, except for ISS ≥ 50. The large difference for the ISS ≥ 50 group is likely due to that the small number of patients makes the estimated effect more uncertain, which is shown by the relatively large confidence intervals. Both the complete case and imputed data analyses show an estimated odds ratio for trauma center care below 0.3 for the ISS ≥ 50 group, and we consider this to be the best estimate from this study (i.e. > 70% mortality reduction), although it should still be considered quite uncertain. During the study period, national trauma alert criteria were developed and implemented in 2016 [30], which decreased the number of triggered trauma team activations and, the number of patients with NISS < 15 reported into the registry. The majority of included patients, 57%, are from four hospitals: Karolinska University Hospital, NÄL (Norra Älvsborgs Länssjukhus) Hospital, Skåne University Hospital and Sahlgrenska University Hospital, which may bias the results. Notably, the northernmost regional trauma center, University Hospital of Umeå, have reported very few patients to SweTrau (*n* = 55 in our final sample). This is an important region to study since the University Hospital of Umeå covers the largest geographical area of all regional trauma centers in Sweden, and it would have been interesting to study the issue of long transportation times within this region.

This study shows that the potential survival benefit for treating patients at Sweden’s regional trauma centers is substantial, from a 41% decrease in mortality risk for all injured patients (ISS ≥ 1) to > 70% reduction for the most severely injured (ISS ≥ 50), regardless of transportation time. Due to a relatively large sample size, reasonably good coverage of Swedish hospitals and confidence intervals with large margins to a neutral effect of specialized care, the results are likely applicable to the whole trauma population in Sweden. Considering the facts that there exists no national transportation destination policy for transporting major trauma patients directly to trauma centers and that a trauma center cannot be reached quickly outside metropolitan areas (Fig. 1), our findings motivate a critical review and possible reorganization of the national trauma system with strategically placed helicopters and trauma centers, possibly similar to the transformation completed in e.g. England that lowered mortality [7].

## Conclusion

We found a potentially substantial survival benefit for trauma patients treated at trauma centers in Sweden, which increased with higher injury severity scores. Further research is needed to identify the characteristics of patients in most need of specialized care and the rate of preventable deaths in Sweden.

## References

[1] World Health Organization (2014). Injuries and violence: the facts, 2014.

[2] Drake SA, Holcomb JB, Yang Y, Thetford C, Myers L, Brock M (2020). Establishing a regional trauma preventable/potentially preventable death rate. Ann Surg..

[3] Ray JJ, Meizoso JP, Satahoo SS, Davis JS, Haren RMV, Dermer H (2016). Potentially preventable prehospital deaths from motor vehicle collisions. Traffic Inj Prev..

[4] Haas B, Gomez D, Zagorski B, Stukel TA, Rubenfeld GD, Nathens AB (2010). Survival of the fittest: the hidden cost of undertriage of major trauma. J Am Coll Surg.

[5] Haas B, Stukel TA, Gomez D, Zagorski B, De Mestral C, Sharma SV (2012). The mortality benefit of direct trauma center transport in a regional trauma system: a population-based analysis. J Trauma Acute Care Surg..

[6] MacKenzie EJ, Rivara FP, Jurkovich GJ, Nathens AB, Frey KP, Egleston BL (2006). A national evaluation of the effect of trauma-center care on mortality. N Engl J Med.

[7] Moran CG, Lecky F, Bouamra O, Lawrence T, Edwards A, Woodford M (2018). Changing the system—major trauma patients and their outcomes in the NHS (England) 2008–17. EClinicalMedicine..

[8] Celso B, Tepas J, Langland-Orban B, Pracht E, Papa L, Lottenberg L (2006). A systematic review and meta-analysis comparing outcome of severely injured patients treated in trauma centers following the establishment of trauma systems. J Trauma.

[9] Sasser S, Hunt R, Faul M, Sugerman D, Pearson W, Theresa D (2012). Guidelines for field triage of injured patients—recommendations of the National Expert Panel on field triage, 2011. MMWR..

[10] American College of Surgeons Committee on Trauma (ACS-COT) (2014). Resources for optimal care of the injured patient.

[11] Baker SP, O’Neill B, Haddon W, Long WB (1974). The injury severity score: a method for describing patients with multiple injuries and evaluating emergency care. J Trauma.

[12] Candefjord S, Buendia R, Caragounis E-C, Sjöqvist BA, Fagerlind H (2016). Prehospital transportation decisions for patients sustaining major trauma in road traffic crashes in Sweden. Traffic Inj Prev..

[13] Fagerlind H, Harvey L, Candefjord S, Davidsson J, Brown J (2019). Does injury pattern among major road trauma patients influence prehospital transport decisions regardless of the distance to the nearest trauma centre?—a retrospective study. Scand J Trauma Resusc Emerg Med..

[14] Holst J, Perman S, Capp R, Haukoos J, Ginde A (2016). Undertriage of trauma-related deaths in U.S. Emergency Departments. Western J Emerg Med..

[15] Xiang H, Wheeler KK, Groner JI, Shi J, Haley KJ (2014). Undertriage of major trauma patients in the U.S. Emergency Departments. Am J Emerg Med..

[16] Rehn M, Lossius HM, Tjosevik KE, Vetrhus M, Østebø O, Eken T (2012). Efficacy of a two-tiered trauma team activation protocol in a Norwegian trauma centre. Br J Surg.

[17] Kodadek LM, Selvarajah S, Velopulos CG, Haut ER, Haider AH (2015). Undertriage of older trauma patients: is this a national phenomenon?. J Surg Res.

[18] Newgard CD, Zive D, Holmes JF, Bulger EM, Staudenmayer K, Liao M (2011). A Multisite Assessment of the American College of Surgeons Committee on trauma field triage decision scheme for identifying seriously injured children and adults. J Am Coll Surg.

[19] Totten AM, Cheney TP, O’Neil ME, Newgard CD, Daya M, Fu R, et al. Physiologic Predictors of Severe Injury: Systematic Review. 5600 Fishers Lane Rockville, MD 20857: Agency for Healthcare Research and Quality, U.S. Department of Health and Human Services; 2018 Apr p. 1–619. Report No.: Number 205, AHRQ Publication No. 18-EHC008-EF. www.ahrq.gov.

[20] Fitzharris M, Stevenson M, Middleton P, Sinclair G (2012). Adherence with the pre-hospital triage protocol in the transport of injured patients in an urban setting. Injury.

[21] Newgard CD, Fu R, Lerner EB, Daya M, Jui J, Wittwer L (2017). Role of Guideline Adherence in Improving Field Triage. Prehosp Emerg Care..

[22] Augenstein JS, Digges KH, Lombardo LV, Perdeck EB, Stratton JE, Malliaris AC (1995). Occult abdominal injuries to airbag-protected crash victims: a challenge to trauma systems. J Trauma.

[23] Schoell SL, Doud AN, Weaver AA, Talton JW, Barnard RT, Winslow JE (2017). Characterization of the occult nature of injury for frequently occurring motor vehicle crash injuries. Accid Anal Prev.

[24] Berwick D, Embrey E, Goldkind SF, Haider A, Holcomb JB, James BC (2016). A national trauma care system: integrating military and civilian trauma systems to achieve zero preventable deaths after injury.

[25] Kristiansen T, Søreide K, Ringdal KG, Rehn M, Krüger AJ, Reite A (2010). Trauma systems and early management of severe injuries in Scandinavia: review of the current state. Injury.

[26] Brattström O. SweTrau Svenska Traumaregistret Årsrapport 2017. Swedish Surgical Society; 2018 p. 1–38. http://rcsyd.se/swetrau/dokument.

[27] Ringdal KG, Coats TJ, Lefering R, Di Bartolomeo S, Steen PA, Røise O, et al. The Utstein Trauma Template for Uniform Reporting of Data Following Major Trauma: Data Dictionary. Version 1.1.1. European Trauma Registry Network. 2008.

[28] Ringdal KG, Coats TJ, Lefering R, Di Bartolomeo S, Steen PA, Roise O (2008). The Utstein template for uniform reporting of data following major trauma: a joint revision by SCANTEM, TARN, DGU-TR and RITG. Scand J Trauma Resusc Emerg Med..

[29] Osler T, Baker SP, Long W (1997). A modification of the injury severity score that both improves accuracy and simplifies scoring. J Trauma.

[30] Linder F, Holmberg L, Bjorck M, Juhlin C, Thorbjornsen K, Wisinger J (2019). A prospective stepped wedge cohort evaluation of the new national trauma team activation criteria in Sweden—the TRAUMALERT study. Scand J Trauma Resusc Emerg Med..

[31] Chow S-C, Shao J, Wang H, Lokhnygina Y (2017). Sample Size Calculations in Clinical Research.

[32] Demetriades D, Martin M, Salim A, Rhee P, Brown C, Chan L (2005). The effect of trauma center designation and trauma volume on outcome in specific severe injuries. Ann Surg.

[33] Twijnstra MJ, Moons KGM, Simmermacher RKJ, Leenen LPH (2010). Regional trauma system reduces mortality and changes admission rates: a before and after study. Ann Surg.

[34] DuBose JJ (2008). Effect of trauma center designation on outcome in patients with severe traumatic brain injury. Arch Surg.

[35] Curtis K, Chong S, Mitchell R, Newcombe M, Black D, Langcake M (2011). Outcomes of severely injured adult trauma patients in an Australian Health Service: does Trauma Center level make a difference?. World J Surg.

[36] Jonsson J, Lubbe N, Strandroth J, Thomson R. The Effect of Advanced Automatic Collision Notification (AACN) on Road Fatality Reduction in Sweden. FAST-zero 2015, September 9–11, Gothenburg, Sweden. 2015.

[37] Campbell-Furtick M, Moore BJ, Overton TL, Laureano Phillips J, Simon KJ, Gandhi RR (2016). Post-trauma mortality increase at age 60: a cutoff for defining elderly?. Am J Surg..

[38] Gaffley M, Weaver AA, Talton JW, Barnard RT, Stitzel JD, Zonfrillo MR (2019). Age-based differences in the disability of extremity injuries in pediatric and adult occupants. Traffic Inj Prev..

[39] Copes WS, Champion HR, Sacco WJ, Lawnick MM, Keast SL, Bain LW (1988). The injury severity score revisited. J Trauma..

[40] Marshall LF, Becker DP, Bowers SA, Cayard C, Eisenberg H, Gross CR (1983). The National Traumatic Coma Data Bank—Part 1: design, purpose, goals, and results. J Neurosurg.

[41] Rimel RW, Giordani B, Barth JT, Boll TJ, Jane JA (1981). Disability caused by minor head injury. Neurosurgery..

[42] Seabold S, Perktold J. Statsmodels: Econometric and Statistical Modeling With Python. In: Proceedings of the 9th Python in Science Conference. Austin, Texas; 2010. http://conference.scipy.org/proceedings/scipy2010/pdfs/seabold.pdf.

[43] Kojima M, Endo A, Shiraishi A, Otomo Y (2019). Age-related characteristics and outcomes for patients with severe trauma: analysis of Japan’s nationwide trauma registry. Ann Emerg Med.

[44] Eid HO, Abu-Zidan FM (2015). New injury severity score is a better predictor of mortality for blunt trauma patients than the injury severity score. World J Surg.

[45] Palmer CS, Gabbe BJ, Cameron PA (2016). Defining major trauma using the 2008 Abbreviated Injury Scale. Injury.

[46] Brown E, Tohira H, Bailey P, Fatovich D, Pereira G, Finn J (2019). Longer prehospital time was not associated with mortality in major trauma: a retrospective cohort study. Prehosp Emerg Care..

[47] Newgard CD, Meier EN, Bulger EM, Buick J, Sheehan K, Lin S (2015). Revisiting the “Golden Hour”: an evaluation of out-of-hospital time in shock and traumatic brain injury. Ann Emerg Med.

[48] Chen X, Guyette FX, Peitzman AB, Billiar TR, Sperry JL, Brown JB (2019). Identifying patients with time-sensitive injuries: association of mortality with increasing prehospital time. J Trauma Acute Care Surg..

[49] Osterwalder JJ (2002). Can the “Golden Hour of Shock” safely be extended in blunt polytrauma patients? Prospective cohort study at a level I hospital in Eastern Switzerland. Prehosp Disaster Med..

[50] Ryb GE, Dischinger P, Cooper C, Kufera JA (2013). Does helicopter transport improve outcomes independently of emergency medical system time?. J Trauma Acute Care Surg..

